# Bulleyaconitine A Exerts Antianxiety and Antivisceral Hypersensitivity Effects

**DOI:** 10.3389/fphar.2020.00328

**Published:** 2020-03-19

**Authors:** Sheng-Nan Huang, BeiBei Yang, Le Ma, Lan-Ting Huang, Pei-Jun Ju, Jinbao Wei, Usman Ali, Yong-Xiang Wang, Jinghong Chen

**Affiliations:** ^1^Shanghai Jiao Tong University School of Medicine, Shanghai Key Laboratory of Psychotic Disorders, Shanghai Mental Health Center, Shanghai, China; ^2^Shanghai Jiao Tong University School of Pharmacy, Shanghai, China

**Keywords:** Bulleyaconitine A (BAA), antianxiety, antivisceral pain, irritable bowel syndrome, microglia, dynorphin A

## Abstract

Visceral pain is one of the leading causes for abdominal pain in gastroenterological diseases and is still hard to treat effectively. Bulleyaconitine A (BAA) is an aconitine analog and has been used for the treatment of pain. Our previous work suggested that BAA exerted analgesic effects on neuropathic pain through stimulating the expression of dynorphin A in spinal microglia. Here, we investigated the inhibitory effect of BAA on visceral pain and examined whether the expression of dynorphin A in spinal microglia was responsible for its effects. We found that BAA produced significant antivisceral pain effect induced by acetic acid through stimulating dynorphin A expression in spinal microglia. In addition, anxiety and chronic visceral pain are highly prevalent comorbid conditions in clinical research, which is still a problem to be solved. We also aimed to evaluate the effects of BAA on anxiety. A comorbidity model with characteristics of both chronic visceral pain and anxiety was developed by colorectal injection of 2,4,6-trinitrobenzene sulfonic acid and the induction of heterotypic intermittent chronic stress protocol. In comorbid animals, BAA exerted great antianxiety effects. Meanwhile, the antianxiety mechanism of BAA was different with the antivisceral pain mechanism of BAA. In conclusion, our study demonstrated, for the first time, that BAA exerted marked antivisceral pain and antianxiety effects, which expands the analgesic spectrum and clinical application of BAA. Furthermore, it also it provides a better guidance for the clinical use of BAA.

## Introduction

Irritable bowel syndrome (IBS) is usually demonstrated by recurrent symptoms of visceral pain or discomfort associated with alterations in bowel evacuation habits. IBS is the most common type of disease among functional gastrointestinal diseases characterized by chronic abdominal intermittent or continuous pain, and changes in bowel evacuation habits (Qu et al., 2013; Winston et al., 2014; Zhao et al., 2018). The pathogenesis of IBS is poorly understood and remains a very challenging question for gastrointestinal researchers and clinicians. Visceral pain, caused by noxious stimulation that activates pain receptors in the internal organs, is one of the most common pains in clinical research. Chronic visceral pain is one of the major kind of abdominal pain caused by gastrointestinal diseases such as IBS, inflammatory bowel disease (IBD), and functional gastrointestinal disorder (FD), etc. (Wouters, 2014; Du et al., 2018). An effective and advanced treatment stratagies for patients with visceral pain are needed for the comfort and ease of humanity (Zhang et al., 2014; Jiang et al., 2018).

Most recently, it has been reported that there exists a high degree of comorbidity between visceral pain diseases and psychological disorders (such as anxiety and depression). The potential link between visceral pain and psychological disorders was stress that worsened the symptoms of patients (Enck et al., 2016), as stress, anxiety and depression are associated with the upregulation of the inflammatory activity (Chen et al., 2014; Zhang et al., 2019). In addition, it has been suggested that psychological disorders such as anxiety were often accompanied by chronic pain (Vidor et al., 2014; Gui et al., 2016; Johnson and Greenwood-Van Meerveld, 2016; Yang et al., 2019). There is still a lack of clinically effective treatments for visceral pain and its comorbid psychological disorders.

Bulleyaconitine A (BAA) is a diterpene alkaloid isolated from the unique medicinal plant of Yunnan, China and is an aconitine-like alkaloid. It has been reported that BAA exerts significant analgesic activities and strong antiinflammatory effects (Zhu et al., 2015; Huang et al., 2016; Li et al., 2016; Xie et al., 2018). In the 1980s, BAA was approved by the State Food and Drug Administration (SFDA) to treat chronic pain symptoms such as arthritis, lower back pain, and lumbar muscle strain (Tang et al., 1986; Wang et al., 2007). It was reported that BAA exerted analgesic effects in several pain models of rats such as the neuropathic pain model, bone cancer pain mode, and formalin-induced tonic pain mode (Zhu et al., 2015; Li et al., 2016; Xie et al., 2018). However, the effects of BAA on visceral pain and its mechanism have not been fully understood yet. Some reports demonstrated that the analgesic effects of BAA were related to voltage-dependent sodium (Nav) channels such as Nav 1.3, Nav 1.7, and Nav 1.8 sodium channels (Brochu et al., 2006; Samad et al., 2013). Previously, we have found that BAA significantly blocked neuropathic pain by stimulating dynorphin A expression in spinal microglia. In the current study, we further explored the effect and mechanism of BAA on visceral pain, expanding our knowledge of the analgesic scope of BAA. We hypothesized that BAA exerted great antivisceral pain effects. In addition, we also investigated the effects of BAA on the bowel habits of 2,4,6-trinitrobenzene sulfonic acid (TNBS)–induced chronic visceral pain to explore the role of BAA in IBS. Our data demonstrated that BAA alleviated the bowel habits in rats with visceral pain and had good potential for treating IBS. Moreover, to further expand the clinical use of BAA and find more effective ways to treat comorbid anxiety in visceral pain, we examined the effects of BAA on anxiety and investigated whether BAA’s mechanism on anxiety was the same as BAA’s analgesic mechanism. Our study suggested that BAA produced a significant anxiolytic effect.

Some research suggested that the chronic stress model and water avoidance stress (WAS) induced anxiety-like behaviors and visceral hypersensitivity (VHS) (Schoenfeld, 2016). In our present research, intracolonic perfusion of TNBS was used to induce neonatal colonic inflammation and exacerbate the development of chronic VHS in rats. Heterotypic intermittent chronic stress (HeICS) application in adult rats was used to aggravate visceral pain and cause anxiety behaviors.

## Materials and Methods

### Drugs and Reagents

BAA and minocycline were purchased from Zelang Bio-Pharmaceutical (Nanjing, China) and Northeast Pharmaceuticals Group (Shenyang, China). Nor-binaltorphimine dihydrochloride (nor-BNI) and 5′-Guanidinonaltrindole (5′-GNTI) were obtained from Abcam (Cambridge, United Kingdom) and Sigma-Aldrich (St. Louis, MO, USA). Dynorphin A (1−17) with peptide sequences of YGGFLRRIRPKLKWDNQ was synthesized by Dan Gang Peptides Co. (Hangzhou, China) with purity not less than 98%. The rabbit polyclonal antibodies neutralizing dynorphin A were purchased from Phoenix Pharmaceuticals (Burlingame, CA, USA), with specificity to dynorphin A (100%), but not to dynorphin B (0%), β-endorphin (0%), α-neo-endorphin (0%), or leu-enkephalin (0%) according to the manufacturer’s descriptions. Its specificity was also validated by the antigen absorption test from other laboratories (Wakabayashi et al., 2010; Yamada et al., 2013). All of the reagents and drugs were diluted or dissolved in 0.9% normal saline or artificial cerebrospinal fluid (ACSF).

### Animals

Female Sprague-Dawley pup rats with a nurturing mother were obtained from Qianbi Biotechnology Company (Shanghai, China). The pup rats were weaned at 22 days of age and were then housed three per cage. The animals were housed in a controlled environment (at standard room temperature (22°C ± 2°C), under conditions of a 12/12-hr reversed light-dark cycle (7:00 am−7:00 pm), receiving water and food ad libitum. The research protocols were approved by the Experimental Animal Committee of Shanghai Jiao Tong University School of Medicine and performed following the Animal Care Guidelines of National Institutes of Health.

### Method for Establishing Acute Visceral Pain Model

Acetic acid writhing experiment was used to establish the acute visceral pain model. Rats were intraperitoneally injected with 1% v/v acetic acid solution (10 ml/kg) and then rats were placed individually in transparent cages (Ghia et al., 2004). The number of acetic acid-induced writhing was counted within 20 min. For the purpose of scoring, a writhe was indicated by simultaneous stretching of the abdomen or at least one hind limb. The reduction in the number of writhes compared to the control group was considered as an evidence of analgesic effect.

### The Single Intrathecal Injection Method

The single intrathecal injection method was undertaken as described previously (Mestre et al., 1994; Mamet et al., 2014). In brief, rats were anesthetized with 2% isoflurane, and kept the lumbar region of the back and lateral surface of the left thigh shaved. A 50-μl Hamilton syringe with an attached 27-gauge needle was inserted between L5 and L6 until the intrathecal space was reached as indicated by tail twitch, then the rats were allowed to recover. Drug testing started 1 week after spinal nerve ligation. Rats received an intrathecal injection of normal saline, blank serum, or dynorphin A antiserum administered 1.5 h before the acetic acid injection. Dynorphin A antiserum (1:10 dilution, 10 μl) or blank serum or normal saline were injected, followed by a 15-μl saline flush.

### Induction of Neonatal Colonic Inflammation

The neonatal pup rats were mildly anesthetized with 2% isoflurane and then were intraluminal administrated with 2,4,6-trinitrobenzene sulfonic acid (TNBS, Sigma Aldrich, MO, USA) (TNBS about 2.86 mg for a 22-g pup, dissolved in 200 μl of the 10% ethanol/90% saline solution) on postnatal day 10, through a catheter into the colon 2 cm from the anus. Rats in the control group were perfused with 200 μl of normal saline. For preventing the leakage from colons, the pups were put in a posture with the head down and anus closed for approximately 2 min.

### HeICS Protocol

HeICS protocol was used to aggravate visceral pain and induce anxiety behaviors in rats (Posserud et al., 2004; Zhang et al., 2014; Winston and Sarna, 2016). HeICS protocol involved daily and multiple stressors at unpredictable times. In this experiment, 4 randomly arranged types of stressors: cold restraint stress(4°C, 45 min), forced swimming stress(25°C ± 1°C, 20 min), WAS (120 min), electricity foot shock (0.5 mA, electricity shock for 10 s, 1 min interval, repeat 30 times) were applied to adult rats in a variable schedule for two consecutive weeks (twice daily) (**Supplementary Table 1**). For cold restraint stress, rats were restrained in a tethered container (diameter, 5 cm × 10 cm high) which was placed in a refrigerator at 4°C for 45 min. For forced swimming stress, rats were forced to swim for 20 min in a plastic container (diameter, 21 cm × 55 cm high) filled with water (23°C). For WAS, rats were placed for 2 h. on a cylinder (diameter, 6 cm × 15 cm high) as an island in the middle of a plastic container (60 cm × 60 cm × 60 cm) filled with water within 2 ~ 3 cm from the top at room temperature. For electricity foot shock, the rat was placed in the fear conditioning box (15 cm high × 16 cm wide × 20 cm long). The current was 0.5 mA for a continuous 10 sec, 1 min interval, 30 times. Cycle the four kinds of stress experiments were cycled to the same rat in sequence and a stress experiment was conducted in the morning and afternoon respectively. The control group rats were kept undisturbed unless changing cages.

### Elevated Plus Maze Test

Anxiety-like behavior of the rats was tested using the elevated plus maze (EPM) paradigm. The EPM was undertaken as described previously (Costa et al., 2014). The device contained one center platform (5 cm × 5 cm), two closed arms and two open arms (30 cm × 5 cm) elevated around 50 cm above the floor. The test was conducted under dim light conditions (50 ~ 60 Lux) between 8:00 am and 12:00 pm. Before the test, rats were put in the testing room and adapted to the room environment for about 30 min. Then rats were put in the EPM apparatus and allowed to explore for 5 min freely. At the beginning, rats were placed individually in the central platform facing an open arm. The total time spent in the open arms was recorded and analyzed using a camera overhead as an index of anxious behaviors (Ethovision XT, Noldus Information Technology, VA, USA). The total distance travelled in the EPM apparatus was recorded and analyzed as a measure of locomotor activity of the rats.

### Open Field Test

The open field test (OFT) was undertaken as described previously (Kuniishi et al., 2017). The device contained a square arena (100 cm × 100 cm) surrounded by walls (40 cm high), which was divided into 16 equal squares and its central part occupied the four squares in the middle. The test was conducted under dim light conditions (50 ~ 60 Lux) between 8:00 am and 12:00 pm. Before the test, rats were brought into the testing room and adapted to room environment for about 30 min. Then rats were put in OFT apparatus and allowed to explore freely for 5 min. A video camera overhead was used to record the behavioral responses of rats during the test. The total time spent in the central area was recorded and analyzed as an index of anxious behaviors (Ethovision XT, Noldus Information Technology, VA, USA). The total distance travelled in OFT was recorded and analyzed as a measure of the locomotor activity of the rats.

### Data Analysis

All data are presented as mean ± standard error of means (SEM). Two-tailed Student’s *t* test or one-way or two-way ANOVA followed by Fisher *post hoc* analysis were used for comparison of means. P < 0.05 was considered statistically significant in all cases.

## Results

### BAA Dose-Dependently Produced Antiacute Visceral Pain, Which Was Inhibited by Minocycline, Dynorphin A Antiserum, and Nor-BNI

The analgesic effects of BAA on acute visceral pain were examined in three groups of rats by PWT (Paw withdrawal threshold), which received a single subcutaneous injection of normal saline (1 ml/kg), BAA (30 μg/kg, dissolved in normal saline, 1 ml/kg) and BAA (90 μg/kg, dissolved in normal saline). One hour after saline or BAA injection, rats were intraperitoneal injected with 1% v/v acetic acid solution (10 ml/kg).

To test whether BAA produced antivisceral pain effect through microglia, the microglia inhibitor-minocycline was applied. Rats were pretreated with minocycline (intraperitoneal injection, 30 mg/kg, 0.1 ml/kg) or saline 2 h before the injection of acetic acid. BAA (subcutaneous injection, 90 μg/kg) or saline was administrated 1 h before the acetic acid injection. To test whether BAA produced an analgesic effect on visceral pain through stimulating dynorphin A expression, dynorphin A antiserum was applied. Rats received an intrathecal injection of normal saline, blank serum or dynorphin A antiserum (1:10 dilution, 10 μl) administered 1.5 h before the acetic acid injection. Then, BAA (subcutaneous injection, 90 μg/kg) or saline was applied 1 h before acetic acid injection. To test whether BAA produced antiacute visceral pain effect through κ-opioid receptors, the κ-opioid receptors inhibitor nor-BNI was used. Nor-BNI (subcutaneous injection, 10 mg/kg) or saline (subcutaneous injection, 1 ml/kg) was applied in rats 2 h before acetic acid injection. Then, BAA (subcutaneous injection, 90 μg/kg) or saline was applied 1 h before acetic acid injection. The number of acid-induced writhes was counted within 20 min.

**Figure 1:**
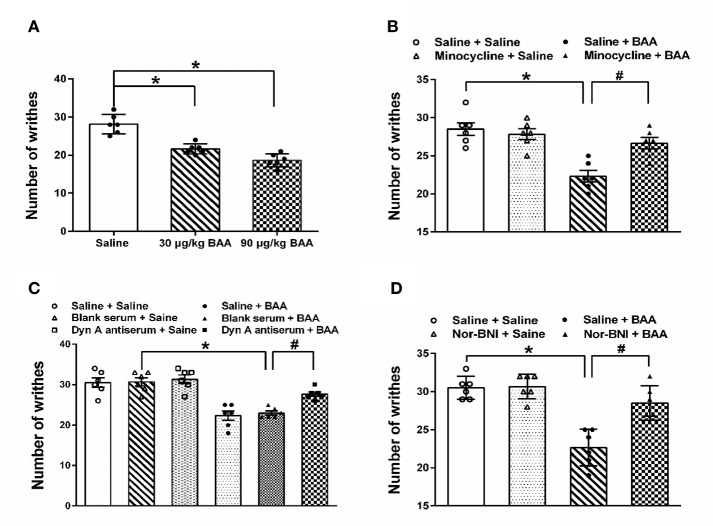
The antivisceral pain effect and the mechanism of Bulleyaconitine A (BAA). **(A)** BAA significantly produced antivisceral pain effect (n = 6 each; one-way ANOVA; ^*^P < 0.05. **(B)** Minocycline blocked the effect of BAA on acute visceral pain in rats (n = 6 each; one-way ANOVA; ^*^P < 0.05; ^#^*P* < 0.05). **(C)** Intrathecal injection of dynorphin A antiserum inhibited the effect of BAA on acute visceral pain in rats (n=6 each; one-way ANOVA; ^*^P < 0.05; ^#^*P* < 0.05). **(D)** Nor-binaltorphimine dihydrochloride (Nor-BNI) blocked the effect of BAA on acute visceral pain in rats (n = 6 each; one-way ANOVA; ^*^P < 0.05; ^#^*P* < 0.05). The data are presented as means ± standard error of means (SEM).

As shown in **Figure 1A**, both 30 and 90 μg/kg BAA produced a significant antiacute visceral pain effect. As shown in **Figure 1B**, microglia inhibitor minocycline inhibited the analgesic effect of BAA on acute visceral pain, while minocycline alone did not influence the number of writhes. The results show that BAA produced antivisceral pain effect through microglia. As shown in **Figure 1C**, the intrathecal injection of dynorphin A antiserum blocked the analgesic effect of BAA on acute visceral pain without changing the number of writhes, which suggested that BAA exerted antivisceral pain effect by activating dynorphin A in spinal. As shown in **Figure 1D**, κ opioid receptors inhibitor nor-BNI inhibited the antivisceral pain effect of BAA and the application of nor-BNI alone did not influence the number of writhes, which demonstrated that BAA produced antivisceral pain effect through κ opioid receptors.

### The Anxiety State Was Significantly Enhanced After HeICS in Rats

**Figure 2:**
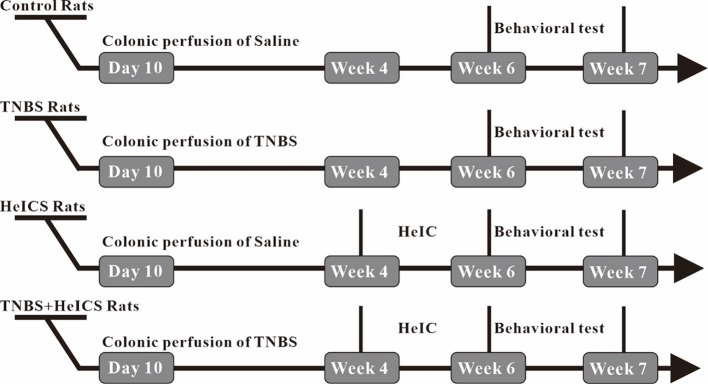
Timeline of chronic visceral pain and anxiety-like behaviors induction experiments.

To investigate the antianxiety effect of BAA in the chronic visceral pain-anxiety comorbidity model and anxiety model, we first induced the anxiety-like behaviors in rats through applying the HeICS protocol (Posserud et al., 2004; Mamet et al., 2014). Rats tagged as TNBS rats (TNBS treated), HeICS rats (HeICS treated), and TNBS + HeICS rats (TNBS followed by HeICS treated) as shown the schematic diagram (**Figure 2**), explaining the whole experimental schedule.

**Figure 3:**
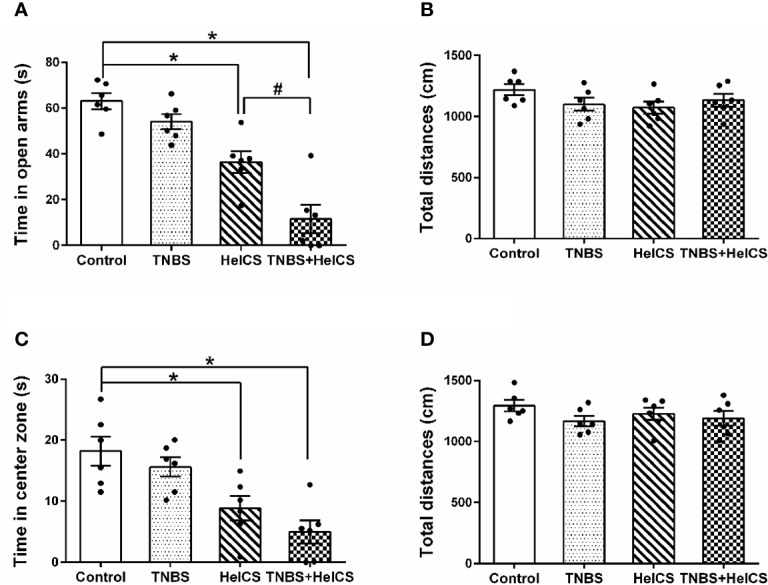
The effects of 2,4,6-trinitrobenzene sulfonic acid (TNBS), heterotypic intermittent chronic stress (HeICS), and TNBS + HeICS application on the anxiety-like behaviors in the elevated plus maze (EPM) test and open field test (OFT). **(A)** Bars showing that both HeICS and TNBS + HeICS application reduced time spent in open arms of the EPM significantly compared with the control group (n = 6 each; one-way ANOVA; ^*^P < 0.05; ^#^*P* < 0.05). **(B)** Bars showing that there were no significant differences of average total distance in the EPM (n = 6 each; one-way ANOVA). **(C)** Bars showing that both HeICS and TNBS + HeICS application observably reduced time spent in center zone compared with the control group (n = 6 each; one-way ANOVA; ^*^P < 0.05). **(D)** Bars showing there were no significant differences of average total distance in the OFT (n = 6 each; one-way ANOVA). The data are presented as means ± standard error of means (SEM).

To test whether the anxiety-like behaviors were induced in rats after HeICS application, EPM paradigm and OFT were carried out. As shown in **Figure 3A**, in the EPM test, both of the HeICS and TNBS + HeICS application significantly reduced time spent in open arms compared with the control group. Moreover, TNBS + HeICS application significantly reduced time spent in open arms compared with HeICS application alone, which suggested that the application of HeICS following TNBS perfusion exerted the anxiety-like behaviors more seriously. As shown in **Figure 3C**, in the OFT, both the HeICS and TNBS + HeICS application significantly reduced time spent in the center zone compared with the control group. In addition, as shown in **Figures 3B, D**, there were no significant differences in the total distance traveled not only in the EPM test but also in the OFT, suggesting that the locomotor activity did not change. These results demonstrated that HeICS application significantly induced the anxiety-like behaviors in rats and TNBS + HeICS application induced the most serious anxiety state in rats. In addition, we also compared the number of action potential (AP) in L5-L6 spinal cord dorsal horn neurons in control rats and TNBS rats. We found that the number of AP in TNBS rats was more than that in control rats, which suggested that the intrinsic excitability of cells in TNBS rats was higher than that in control rats.

### BAA Produced Antianxiety Effects in TNBS+HeICS Rats

**Figure 4:**
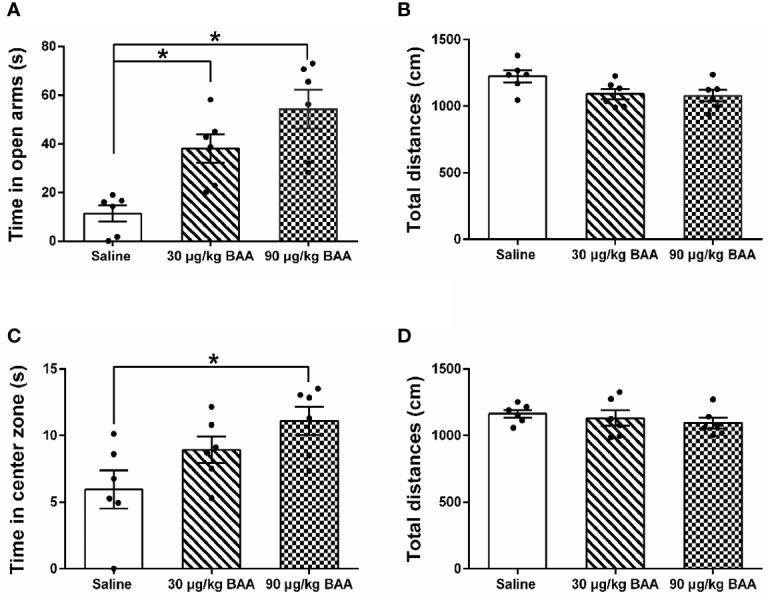
The antianxiety effects of Bulleyaconitine A (BAA) on chronic visceral pain-anxiety comorbidity of 2,4,6-trinitrobenzene sulfonic acid + heterotypic intermittent chronic stress (TNBS+HeICS) rats. **(A**, **C)** The antianxiety effects of BAA on chronic visceral pain-anxiety comorbidity rats in the elevated plus maze (EPM) test and open field test (OFT) (n = 6 each; one-way ANOVA; ^*^P < 0.05). **(B**, **D)** BAA did not influence the total distance whether in the EPM test or in the OFT. The data are presented as means ± standard error of means (SEM).

To investigate the antianxiety effect of BAA in the chronic visceral pain-anxiety comorbidity model, we first studied the effect of BAA in TNBS+HeICS rats. Three groups of TNBS + HeICS rats were with injected normal saline (1 ml/kg), BAA (30 μg/kg), and BAA (90 μg/kg) separately on 1 d (EPM) and 2 d (OFT) after HeICS. As shown in **Figures 4A, C**, BAA significantly increased the time spent in the open arms of EPM and in center zone of OFT, suggesting that BAA reduced the anxiety-like behaviors in chronic visceral pain-anxiety comorbidity rats. In addition, as shown in **Figures 4B, D**, there were no significant differences in the total distance traveled not only in the EPM but also in the OFT, suggesting that subcutaneous administration of 30 μg/kg and 90 μg/kg BAA did not alter locomotor activity in rats.

### BAA Did Not Exert Antianxiety Effects Through Stimulating Dynorphin A Expression

**Figure 5:**
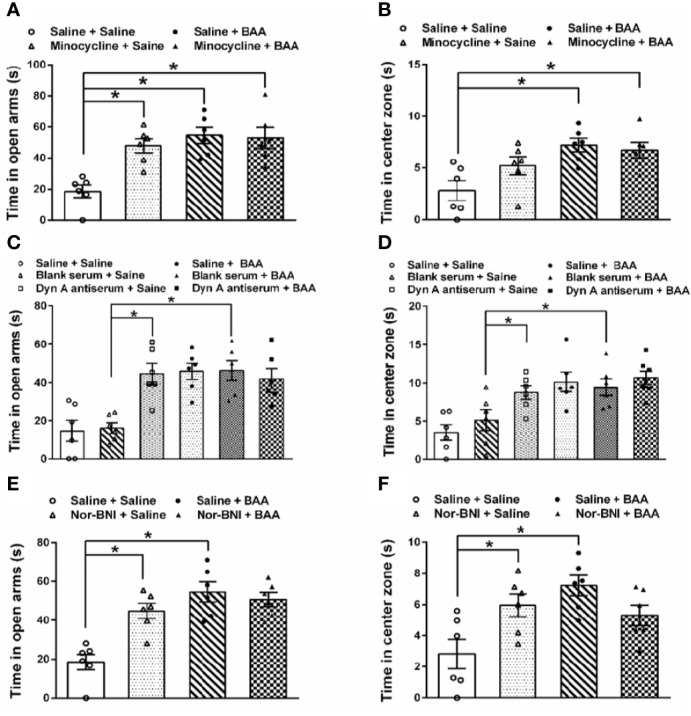
The effects of microglia inhibitor minocycline, dynorphin A antiserum, and κ opioid receptors inhibitor nor-binaltorphimine dihydrochloride (nor-BNI) on the antianxiety effect of Bulleyaconitine A (BAA). **(A)** Bars showing that in the elevated plus maze (EPM) test, both BAA and minocycline produced significantly antianxiety effects on 2,4,6-trinitrobenzene sulfonic acid + heterotypic intermittent chronic stress (TNBS+HeICS) rats (n = 6 each; one-way ANOVA; ^*^P < 0.05). **(B)** Bars showing that in the open field test (OFT), minocycline did not influence the antianxiety effect of BAA (n = 6 each; one-way ANOVA; ^*^P < 0.05). **(C)** Bars showing that both the administration of dynorphin A antiserum and BAA increased time spent in the open arms of the EPM test. (n = 6 each; one-way ANOVA; ^*^P < 0.05. **(D)** Bars showing that both the administration of dynorphin A antiserum and BAA increased time spent in center zone of the OFT (n = 6 each; one-way ANOVA; ^*^P < 0.05. **(E)** Bars showing that in TNBS + HeICS rats, both the injection of nor-BNI or BAA increased time spent in open arms of EPM significantly (n = 6 each; one-way ANOVA; ^*^P < 0.05). Moreover, the administration of BAA and nor-BNI together did not exert more effective antianxiety effects. **(F)** Bars showing that in TNBS + HeICS rats, both the injection of nor-BNI or BAA increased time spent in center zone of OFT (n = 6 each; one-way ANOVA; ^*^P < 0.05). Moreover, the administration of BAA and nor-BNI together did not produce more effective antianxiety effects. The data are presented as means ± standard error of means (SEM).

To investigate whether the antianxiety effect of BAA was also by stimulating dynorphin A expression in spinal microglia, microglia inhibitor minocycline, dynorphin A antiserum, and κ opioid receptors inhibitor nor-BNI were applied. As shown in **Figures 5A, B**, BAA significantly reduced the anxiety-like behaviors in TNBS + HeICS rats whether in the EPM test or in the OFT, while the single intraperitoneal injection of minocycline also decreased the anxiety-like behaviors of rats in the EPM. However, the application of minocycline and BAA together did not produce more effective antianxiety effects in the EPM and OFT. The results showed that the antianxiety mechanism of BAA may not work through microglia and the minocycline also exerted anxiolytic effects. As shown in **Figures 5C, D**, both intrathecal injection of dynorphin A antiserum or subcutaneous injection of BAA alone significantly reduced the anxiety-like behaviors in TNBS + HeICS rats whether in the EPM test or OFT, while the intrathecal injection of blank antiserum alone did not influence the anxiety state of rats. Moreover, the application of dynorphin A antiserum and BAA together did not produce superimposed anxiolytic effects. The results suggested that the anxiolytic mechanism of BAA may not work through stimulating dynorphin A expression and dynorphin A antiserum also exerted great antianxiety effects. As shown in **Figures 5E, F**, BAA significantly reduced the anxiety-like behaviors in TNBS + HeICS rats both in the EPM test and in the OFT, while the subcutaneous injection of nor-BNI also alleviated the anxiety state of TNBS + HeICS rats in the EPM test. However, the application of nor-BNI and BAA together did not produce additional anxiolytic effect in the EPM test. The results suggested that the anxiolytic mechanism of BAA may not work through κ-opioid receptors and the κ-opioid receptor inhibitor nor-BNI also exerted anxiolytic effects. These results above demonstrated that the antianxiety mechanism of BAA was different with the antivisceral pain mechanism of BAA. In addition, the activation of microglia, the stimulation of dynorophin A and the activation of κ-opioid receptors may induce anxiety-like behaviors in rats. The reason for these contradicting results might be that the secretion of dynorphin A stimulated by BAA (approximately 40 pg/mg protein in spinal homogenates) (Li et al., 2016) was not enough to induce anxiety in rats.

### BAA Produced Antianxiety Effects in HeICS Rats

**Figure 6:**
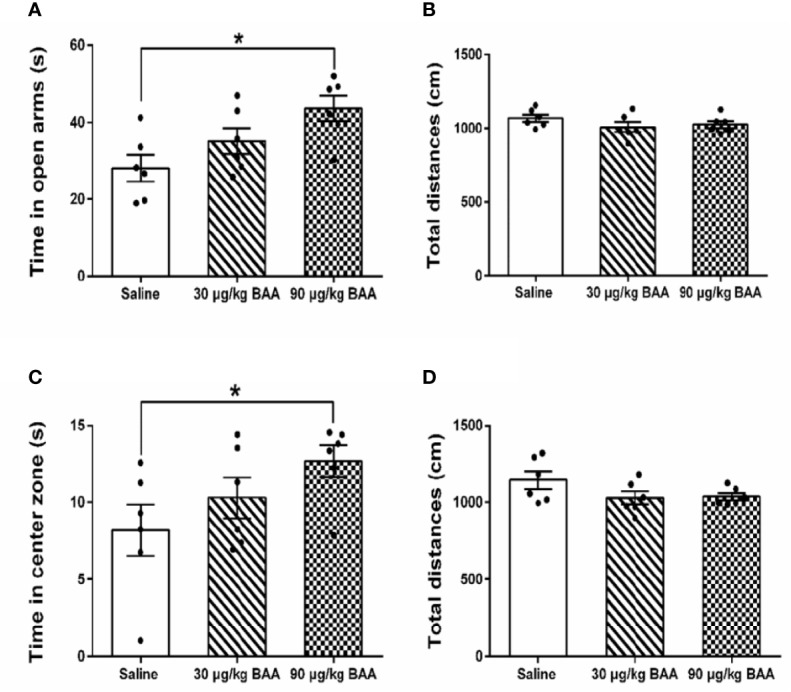
The antianxiety effects of Bulleyaconitine A (BAA) on visceral pain-anxiety comorbidity of heterotypic intermittent chronic stress (HeICS) rats. **(A**, **C)** The antianxiety effects of BAA on anxiety model rats in elevated plus maze (EPM) and open field test (OFT) (n = 6 each; one-way ANOVA; ^*^P < 0.05 90 μg/kg BAA vs. 1 ml/kg Saline). **(B**, **D)** BAA did not influence the total distance whether in EPM or in OFT. The data are presented as means ± standard error of means (SEM).

To further confirm the antianxiety effect of BAA, we next study the effect of BAA in HeICS rats. Three groups of HeICS rats in 4 weeks were injected normal saline (1 ml/kg), BAA (30 μg/kg), and BAA (90 μg/kg) on 1 d (EPM) and 2 d (OFT) after the last stress respectively. As shown in **Figures 6A, B**, 90 μg/kg BAA significantly increased time spent in open arms of EPM without changing the total distances travelled in EPM. Similarly, as shown in **Figures 6C, D**, 90 μg/kg BAA also increased time spent by rats in the center part of OFT without influencing the total distances travelled in OFT. These results suggested 90 μg/kg BAA also produced great antianxiety effects in HeICS rats, which further confirmed the antianxiety effects of BAA.

### BAA Improved the Gastrointestinal Function With Visceral Pain

**Figure 7:**
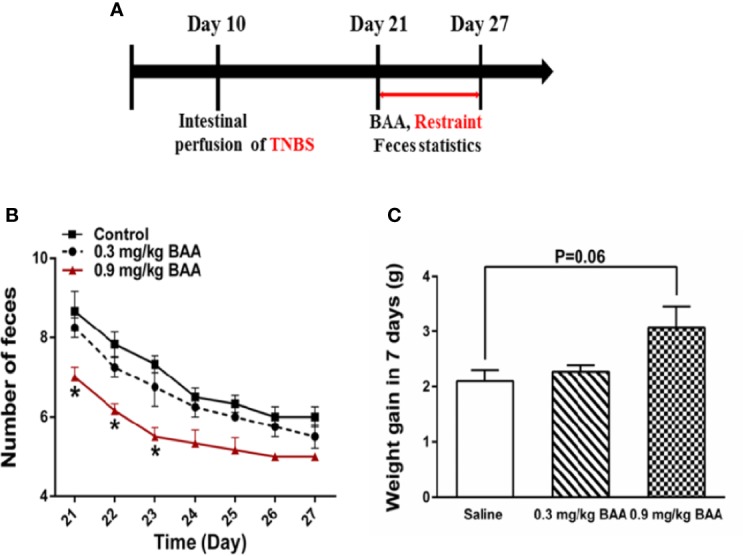
The effects of Bulleyaconitine A (BAA) on the amount of feces and weight gain during puberty in rats with chronic visceral pain. **(A)** Timeline of experimental protocols to induce chronic visceral pain in rats. **(B)** The effect of BAA on the number of bowel movements in rats after 2 h of restraint stress every day (n=6 each; one-way ANOVA; ^*^P < 0.05 0.9 mg/kg BAA vs. Control). **(C)** The effect of BAA on the weight gain of rats during the experiment (n = 6 each; one-way ANOVA). The data are presented as means ± standard error of means (SEM).

To investigate the influence of BAA on bowel habits and gastrointestinal function during puberty in rats with chronic visceral pain, rats were divided into the experimental group and control group. Both groups of rats were perfused with TNBS solution in the colon at 10 day after birth. When the rats were 3 weeks old, rats in the experimental group were intragastrically administered with BAA (0.8 mg/kg, 10 ml/kg) and rats in the control group were intragastrically administered with normal saline (**Figure 7A**). Then two groups of rats were restrained in a tethered container (diameter, 5 cm × 10 cm high) for 2 h to aggravate the chronic visceral pain in rats. Two hours later, the rats were taken out and the amount of feces in the bottles was counted. As shown in **Figures 7B, C**, 0.9 mg/kg BAA reduced the number of feces in rats with chronic visceral pain and the body weight of rats had a larger range of weight gain during the gastrointestinal administration of BAA in the fast growth adolescent period. The results suggested that the gastrointestinal administration of BAA improved bowel habits and gastrointestinal function during puberty in rats with chronic visceral pain.

## Discussion

Visceral pain is usually characterized by inaccurate localization and frequent referred pain (Zhang et al., 2015), which differs significantly in terms of neurological mechanisms. Visceral pain is one of the main causes for abdominal pain and has been an important biological hallmark of pain symptoms clinically (Keszthelyi et al., 2012; Hasler and Owyang, 2013; Camilleri, 2014; Wouters, 2014). It is well known that opioids play an important role in the management of visceral pain and other effective alternatives are still to be discovered. There are many dose-dependent side effects of opioid drugs such as constipation, respiratory symptoms, and depression (Pereira Do Carmo et al., 2009). Despite continuous advances in the treatment of visceral pain, additional research is needed to find a more effective treatment for it. BAA, a diterpenoid alkaloid isolated from *Aconitum Bulleyanum*, belongs to the “aconitine-like” alkaloids and has been used for the treatment of chronic pain for around three decades, but the molecular mechanism is still not fully discovered. There are many contradictory reports about the analgesic mechanism of BAA. Some studies have stated that the blockage of voltage-dependent sodium (Nav) channels such as Nav 1.3, Nav 1.7, and Nav 1.8 sodium channels by BAA produce antinociceptive effects on neuropathic pain in rats (McGaraughty et al., 2008; Samad et al., 2013). The latest research reported that BAA might exert an antineuropathic pain effect by use-dependent blocking tetrodotoxin-sensitive Nav1.3 and Nav1.7 channels in dorsal root ganglion neurons (Xie et al., 2018). Moreover, it was reported that BAA attenuated paclitaxel induced neuropathic pain, which might be related to the inhibition of spinal LTP at C-fiber synapses by inhibiting presynaptic transmitter release (Zhu et al., 2015).

In the present study, we have further expanded the analgesic spectrum of BAA. Our data demonstrated for the first time that BAA produced significant antivisceral pain effect on acute visceral pain induced by acetic acid. More specifically, our current results revealed that BAA also exerted antivisceral pain effects through stimulating dynorphin A in spinal microglia and then dynorphin A acted on κ-opioid receptors, consistent with the analgesic mechanism of BAA on neuropathic pain in our previous research (Li et al., 2016). Dynorphin A is an endogenous opioid neurotransmitter and is produced in the spinal cord and many parts of the brain such as the striatum, hippocampus and the hypothalamus (Mollereau et al., 1999). Previously, we have found that the secretion and localization of dynorphin A was not only in neurons and astrocytes as reported in other studies, but also in microglia by using double immunostaining. In addition, BAA stimulated the expression of dynorphin A (measured by the levels of the prodynorphin and dynorphin A gene expression) in the primary culture of microglia but not in astrocytes or neurons from neonatal and adult rats, even though the latter two types of cells also expressed and secreted dynorphin A (Li et al., 2016). Moreover, in our present research, we have found that the antivisceral pain effect of BAA given subcutaneously was inhibited by microglia inhibitor minocycline, dynorphin A antiserum (intrathecal injection) and κ opioid receptors antagonist nor-BNI. In the current study, we also examined the effect of BAA on the poor bowel habits and gastrointestinal function in rats with chronic visceral pain. We found that BAA reduced the number of feces and increased the range of weight gain in rats with chronic visceral pain. These results clearly demonstrated for the first time that BAA not only produced great antivisceral pain effect but also improved the gastrointestinal function in rats with visceral pain, which suggested BAA might be a potential drug for the clinical treatment of visceral pain and IBS. This study also provided a new basis for BAA to expand its clinical application.

Previously, it was reported that there was a high degree of comorbidity between visceral pain diseases and psychological disorders (such as anxiety and depression). In our present research, we found that BAA produced significant antianxiety effects in rats with chronic visceral pain-anxiety comorbidity. Furthermore, the antianxiety mechanism of BAA was different with the antivisceral pain mechanism of BAA. It has been suggested that chronic stress increased gene expression of some inflammatory factors in the hippocampus such as IL-1β, IL-18, and IL-6. IL-1β is an important mediator of stress-induced anxiety-like behavior and it has been reported that the hippocampal IL-1β mRNA was upregulated in stressed rats (Jones et al., 2018; Wang et al., 2018). More importantly, the mature form of IL-1β as well as its convertase, cleaved caspase-1, were both increased in the hippocampus after exposure to chronic stress(Wang et al., 2018). Based on the previous studies, we believe that the antianxiety effect of BAA might be related to the changes of some inflammatory factors such as IL-1β after HeICS. The results confirmed for the first time that BAA produced a significant antianxiety effect, which further expanded the clinical application of BAA and showed the possibility of BAA treating anxiety in clinical practice.

## Ethics Statement

This study was carried out in accordance with the recommendations of the Experimental Animal Committee of Shanghai Jiao Tong University School of Medicine. The protocol was approved by the Animal Care Guidelines of the National Institutes of Health.

## Author Contributions

Conceived and designed the experiments: JC, Y-XW. Performed the experiments: S-NH, BY, and LM. Analyzed the data: S-NH, P-JJ, L-TH, J-BW, and JC. Preparation of the paper: JC, S-NH, BY, J-BW, UA, and Y-XW.

## Funding

This study was supported in part by grants from the National Natural Science Foundation of China (#81571326 to JC and #81673403 to Y-XW), the Shanghai Industrial Translational Project (#15401901300 to Y-XW), and the Shanghai Key Laboratory of Psychotic Disorders (16-K01 to Y-XW).

## Conflict of Interest

The authors declare that the research was conducted in the absence of any commercial or financial relationships that could be construed as a potential conflict of interest.

The reviewer QH declared a past supervisory role with one of the authors Y-XW to the handling editor.

## Supplementary Material

Supplementary Table 1 HeICS protocol was used to aggravate visceral pain and induce anxiety behaviors in rats. Table 1. Each rat from HeICS group were treated with the validated protocol as above.

## Abbreviations

BAA, Bulleyaconitine A; VHS, Visceral hypersensitivity; IBS, Irritable bowel syndrome; HeICS, Heterotypic intermittent chronic stress; TNBS, 2,4,6-trinitrobenzene sulfonic acid; CRD, Colorectal distension; Nor-BNI, Nor-binaltorphimine dihydrochloride; OFT, Open field test; EPM, Elevated plus maze; AWR, Abdominal withdrawal reflex; PWT, Paw withdrawal threshold.
