# Supplementary material for: Bulleyaconitine A Exerts Antianxiety and Antivisceral Hypersensitivity Effects
Source: Front Pharmacol. 2020 Mar 19;11:328. doi: 10.3389/fphar.2020.00328 (PMC7098429; doi:10.3389/fphar.2020.00328)
Supplement: Supplementary Table 1 — HeICS protocol was used to aggravate visceral pain and induce anxiety behaviors in rats. Table 1. Each rat from HeICS group were treated with the validated protocol as above. [file Table_1.pdf]

Table 1 Protocol of HeICS

Time	The type of stress in the morning	The type of stress in the afternoon
Day1	water avoidance	cold restraint
Day2	forced swimming	electricity foot shock
Day3	cold restraint	forced swimming
Day4	electricity foot shock	water avoidance
Day5	forced swimming	electricity foot shock
Day6	water avoidance	cold restraint
Day7	electricity foot shock	water avoidance
Day8	cold restraint	forced swimming
Day9	water avoidance	cold restraint
Day10	forced swimming	electricity foot shock
Day11	cold restraint	forced swimming
Day12	electricity foot shock	water avoidance
Day13	forced swimming	electricity foot shock
Day14	water avoidance	cold restraint
